# Cancer-related pain in head and neck cancer survivors: longitudinal findings from the Head and Neck 5000 clinical cohort

**DOI:** 10.1007/s11764-024-01554-x

**Published:** 2024-02-29

**Authors:** Iakov Bolnykh, Joanne M Patterson, Sam Harding, Laura-Jayne Watson, Liya Lu, Katrina Hurley, Steve J Thomas, Linda Sharp

**Affiliations:** 1 Population Health Sciences Institute, Newcastle University Centre for Cancer, Newcastle, UK; 2 Liverpool Head and Neck Centre, School of Health Science, University of Liverpool, Liverpool, UK; 3 Speech and Language Therapy Research Unit, Southmead Hospital North Bristol NHS Hospital Trust, Bristol, UK; 4 Speech & Language Therapy, Sunderland Royal Hospital, South Tyneside and Sunderland NHS Foundation Trust, Sunderland, UK; 5 NHS Forth Valley, Stirling, Scotland, UK; 6 Head & Neck 5000 Study, University Hospitals Bristol and Weston NHS Foundation Trust and University of Bristol, Bristol, UK

**Keywords:** HNC, Pain, HN5000, Survivorship

## Abstract

**Purpose:**

Reports suggest pain is common in head and neck cancer (HNC). However, past studies are limited by small sample sizes and design and measurement heterogeneity. Using data from the Head and Neck 5000 longitudinal cohort, we investigated pain over a year post-diagnosis. We assessed: temporal trends; compared pain across HNC treatments, stages, sites and by HPV status; and identified subgroups of patients at increased risk of pain.

**Methods:**

Sociodemographic and clinical data and patient-reported pain (measured by EORTC QLQ-C30 and QLQ-H&N35) were collected at baseline (pre-treatment), 4- and 12- months. Using mixed effects multivariable regression, we investigated time trends and identified associations between (i) clinically-important general pain and (ii) HN-specific pain and clinical, socio-economic, and demographic variables.

**Results:**

2,870 patients were included. At baseline, 40.9% had clinically-important general pain, rising to 47.6% at 4-months and declining to 35.5% at 12-months. HN-specific pain followed a similar pattern (mean score (sd): baseline 26.4 (25.10); 4-months. 28.9 (26.55); 12-months, 17.2 (19.83)). Across time, general and HN-specific pain levels were increased in: younger patients, smokers, and those with depression and comorbidities at baseline, and more advanced, oral cavity and HPV negative cancers.

**Conclusions:**

There is high prevalence of general pain in people living with HNC. We identified subgroups more often reporting general and HN-specific pain towards whom interventions could be targeted.

**Implications for cancer survivors:**

Greater emphasis should be placed on identifying and treating pain in HNC. Systematic pain screening could help identify those who could benefit from an early pain management plan.

**Supplementary Information:**

The online version contains supplementary material available at 10.1007/s11764-024-01554-x.

## Introduction

Head and neck cancer (HNC) is the sixth most common cancer worldwide with a range of challenges related to high burden of disease, late presentation, and poor access to care [1, 2]. Rising incidence has been reported internationally, attributed to human papilloma virus (HPV) infection. While HPV-positive tumours most commonly occur in the oropharynx, some oral cavity and larynx cancers are also HPV-positive [3]. Recent advances in HNC treatment have improved long-term patient survival and the number of people living with and beyond this cancer. This, in turn, has increased the scope for, and interest in, measuring functional and quality of life (QoL) related outcomes in survivors [4]. QoL has been shown to be an independent prognostic factor for survival in many types of cancer, including HNC, which has further emphasised its importance [5, 6].

Pain is a presenting symptom for up to 70% of patients diagnosed with HNC [7]. The head and neck contains many anatomical structures in a confined space with a significant degree of nerve innervation [8]. As the cancer progresses, these nerves become directly stimulated by the tumours and become prone to perineural invasion (PNI) by the HNC, and many of the proteins and genes implicated in PNI are the same proteins involved in pain signalling [9, 10]. Pain experienced by HNC patients is heterogenous and includes spontaneous pain, function-evoked pain, referred pain, widespread pain, and pain suggestive of peripheral neuropathy [10]. Various factors have been shown to impact pain, including the extent of PNI and metastases, tumour size, location, and stage.

Pain can also be a side-effect of HNC treatment. Surgery can lead to neuropathic pain via direct nerve transection. Lymph node dissection in particular, often leads to the unavoidable cutting of sensory nerve branches [11]. Surgery can also lead to chronic pain due to muscle imbalances resulting from the removal of neck muscles [12, 13]. Non-surgical treatments can be responsible for pain; for instance radiotherapy can lead to osteoradionecrosis, or xerostomia, and although not specific to HNC, chemotherapy can result in peripheral neuropathy and mucositis [14, 15].

Cancer-related pain has an important impact on the lives of people with HNC. The pain itself affects basic functions such as eating and swallowing, as well as interpersonal relations while ineffective control correlates with decreased QoL and poor outcomes [1, 16, 17]. Patients with HNC are prescribed opioids more often than those with other cancers, which comes with significant side-effects and risk for dependence [10, 18].

Despite this, the overall prevalence of pain in HNC, and the factors associated with it, are unclear. Most studies are limited by small sample sizes and cross-sectional designs and, although a range of patients characteristics and other factors have been studied for associations with pain, some studies include individuals treated two decades ago and/or include those with cancer at a specific (sub)site within the head and neck [19–21]. The treatment landscape of HNC has also changed dramatically with wider use of minimally invasive surgical techniques, more-precisely targeted radiotherapy and chemotherapy agents with fewer toxic side-effects [22]. Longitudinal studies are limited, so temporal trends are unclear. Furthermore, there is no consistent measurement of pain; for example, some studies report general pain and others HN-specific pain.

Using data from the Head and Neck 5000 (HN5000) prospective, longitudinal, cohort study, we investigated the prevalence of cancer pain (both clinically-important general pain and HN-specific pain) over the first 12 months post-diagnosis. Our objectives were to: (1) assess temporal trends; (2) compare pain across HNC treatments, stages and sites, and by HPV status; and (3) identify subgroups at higher risk of developing pain.

## Materials & methods

### Study population

The HN5000 protocol and population has been described elsewhere [23, 24]. Participants in the study included individuals aged 16 and older with a new primary or suspected HNC from 76 UK centres. The clinical teams at sites excluded individuals who lacked the capacity to give consent or who they felt were too vulnerable to take part in the study.

In the dataset (version 2.1) utilised for the analysis, 5,404 people who consented to participate between April 2011 and December 2014 were included. In our analysis, we only included individuals who had been diagnosed with cancers of the thyroid, salivary gland, hypopharynx, oropharynx, larynx, and/or oral cavity, undergone treatment, and were free of recurrence during follow-up (*N* = 3,779). The small sample of patients with hypopharyngeal cancers were combined with laryngeal cancers for analysis. The full breakdown of the study population can be seen in the flow diagram in Supplementary Fig. 1.

### Procedures

Participants completed a series of questionnaires focusing on health and lifestyle at baseline (shortly after diagnosis but before treatment started), 4 months, and 12 months. The baseline survey included socio-demographic (e.g., income, marital status) and lifestyle (e.g., smoking, alcohol consumption) characteristics and included the EORTC-QLQ-C30 [25], the EORTC-H&N35 [26] and the Hospital Anxiety & Depression Scale (HADS), a validated screening tool for anxiety and depression [27].

A range of information was abstracted from participants’ medical records. This included demographic characteristics such as age and sex, the ICD10 tumour site [28], TNM-stage [29], laterality of primary tumour and serological human papilloma virus (HPV) status at baseline (which was defined as positive where HPV16E6 was > 1000 median fluorescence intensity) [30]. Treatment received by 4 months was also abstracted as well as the information on whether the tumour had recurred by 4 months or 12 months.

The Adult Comorbidity Evaluation (ACE-27) [31] was used to assign a comorbidity status to each participant. A deprivation category was also assigned to each participant’s home address at baseline, based on the English Index of Multiple Deprivation 2010 [32]. Significant depression was defined with a score of ≥ 8 on the HADS depression subscale, following recommendations for detecting depression in people with cancer [33].

### Outcome variables – clinically-important general pain and HN-specific pain

Our study focused on two outcome variables: (i) clinically-important general pain measured using the EORTC-QLQ-C30 and (ii) HN-specific pain measured using the EORTC-QLQ-H&N35. The QLQ-C30 pain subscale includes two questions on general pain (had pain; pain interfered with daily activities) and the H&N35 pain subscale contains four questions focusing more on the head and neck (had pain in the mouth, jaw or throat; had soreness in the mouth), with both questionnaires focusing on pain experienced over the last week. For both subscales, questions were linearly transformed into a score ranging from 0 to 100, with higher scores representing more severe pain. Missing data was treated as recommended by the questionnaire developers [25, 26].

For the QLQ-C30 pain subscale, a score of over ≥ 25/100 indicates the presence of clinically-important pain [34]. We created a binary variable that indicated the presence or absence of clinically-important general pain based on the QLQ-C30 subscale score; the HN-specific pain subscale was treated as a continuous outcome variable.

### Statistical analysis

To be included in relevant analyses, patients had to have completed either the QLQ-C30 pain subscale or the HN-specific pain subscale at baseline (Supplementary Fig. 1). Within the study population (*n* = 3,779), we compared characteristics of those who did, and did not, complete these subscales using chi-square tests.

Amongst those included in analyses, we assessed and compared the proportions with clinically-important general pain and mean scores for HN-specific pain at baseline, 4 months, and 12 months by clinical and socio-demographic characteristics. We used mixed-effects multivariable regression (logistic for general pain, linear for HN-specific pain) to examine temporal trends and factors associated with pain over time. These models allowed us to include all surveys completed by an individual, account for correlations between subjects, and produce robust estimates of error [35].

The following model fitting process was followed first for general pain and then repeated for HN-specific pain. We first examined bivariate associations between candidate predictor variables (socio-demographic, lifestyle and clinical variables (Supplementary Tables 2 and 3)) and the outcome, adjusted for time point. Time point and variables significant at the 5% level were then included in the initial model. Covariables no longer significant at the 5% level in this initial model (assessed using Wald statistics) were then removed. We used Akaike’s Information Criterion (AIC) to compare models with random intercept and random slope with those including random intercept only. Due to the minimal difference between the models, we fitted the least complex option – random intercept with structured covariance. If < 2% of individuals had missing data for a variable, the data was excluded. If the level of missing data was ≥ 2%, an “unknown” category was added to the variable and included in analyses. STATA 17.0 was used for the analysis.

## Results

### Participants

We included 2,870 and 2,855 patients in the general pain and HN-specific pain analyses respectively (Supplementary Fig. 1). There were no differences in age, treatment, tumour site and laterality between those who completed the baseline questionnaire pain subscales and those who did not. There were statistically significant differences in deprivation, comorbidities, and cancer stage; our analysis included fewer deprived and comorbid patients, as well as fewer patients with higher stage cancers.

The demographic characteristics of individuals included in the analyses can be seen in Supplementary Table 1. Of the people included in the analysis datasets, almost two-thirds were male, just over a quarter had stage 1 disease, and almost half had had multimodal treatment. Almost 90% had cancers in the oropharynx (39%), oral cavity (25%) or larynx (25%), sites at which HPV-positive tumours may occur; overall, 61% of patients had HPV-positive disease.

### General pain at each time point

At baseline, 40.9% of patients experienced clinically-important general pain. This figure rose to 47.6% at 4 months and declined to 35.5% (below baseline) at 12 months (Table 1).

**Table 1:** Prevalence of clinically-important pain* at baseline, 4 months, and 12 months, by clinical variables. Number who completed subscale (N), number who scored in range for clinically-important pain (n) and percentages (%)

	Baseline		4 Months		12 Months	
**Explanatory variable**	N (n)	% clinically-important	N (n)	% clinically-important	N (n)	% clinically-important
**Treatment received**^**1**^						
*Surgery*	845 (325)	38.5	752 (293)	39.0	644 (229)	35.6
*Chemoradiotherapy*	787 (332)	42.2	666 (350)	52.6	617 (213)	34.5
*Radiotherapy*	540 (205)	38.0	435 (180)	41.4	395 (139)	35.2
*Surgery and radiotherapy*	421 (191)	45.4	379 (216)	57.0	345 (125)	36.2
*Surgery and chemoradiotherapy*	277 (122)	44.0	237 (135)	57.0	204 (76)	37.3
**Chi2 p value**		0.057		< 0.001		0.960
**Tumour stage**						
*1*	797 (275)	34.5	685 (245)	35.8	590 (189)	32.0
*2*	485 (205)	42.3	426 (197)	46.2	367 (133)	36.2
*3*	393 (159)	40.5	349 (177)	50.7	318 (127)	39.9
*4*	1184 (532)	44.9	1003 (552)	55.0	923 (330)	35.8
**Chi2 p value**		< 0.001		< 0.001		0.114
**Cancer site**						
*Oral cavity*	716 (346)	48.3	655 (310)	47.3	548 (209)	38.1
*Oropharynx*	1123 (484)	43.1	958 (512)	53.4	911 (313)	34.4
*Larynx + Hypopharynx*	713 (254)	35.6	590 (257)	43.6	518 (192)	37.1
*Thyroid*	186 (49)	26.3	155 (46)	29.7	129 (32)	24.8
*Minor and major salivary glands*	132 (42)	31.8	111 (49)	44.1	99 (36)	36.4
**Chi2 p value**		< 0.001		< 0.001		0.056
**HPV16 E6**						
*Positive*	1741 (713)	41.0	1500 (674)	44.9	1318 (479)	36.3
*Negative*	765 (288)	37.7	647 (324)	50.1	617 (194)	31.4
*Unknown*	364 (174)	47.8	322 (176)	54.7	270 (109)	40.4
**Chi2 p value**		0.005		0.002		0.022

*as measured by EORTC QLQC-30

1 “Surgery only” includes 1 patient who had surgery and chemotherapy; “Chemoradiotherapy only” includes 5 patients who only had chemotherapy

**Table 2:** Head and neck specific pain* mean scores (standard deviation (sd)) at baseline, 4 months, and 12 months, by clinical variables. Number of participants who completed subscale (N)

	Baseline		4 Months		12 Months	
**Explanatory variable**	N	Mean (sd)	N	Mean (sd)	N	Mean (sd)
**Treatment received**^**1**^						
*Surgery*	842	23.4 (23.31)	752	15.5 (18.97)	642	13.4 (17.74)
*Chemoradiotherapy*	783	29.5 (25.67)	666	39.3 (26.42)	614	21.2 (21.18)
*Radiotherapy*	538	19.4 (21.62)	434	21.3 (23.73)	393	13.9 (18.59)
*Surgery and radiotherapy*	416	32.5 (28.48)	378	39.2 (28.04)	343	19.7 (19.62)
*Surgery and chemoradiotherapy*	276	30.9 (26.38)	235	29.6 (26.40)	204	19.7 (21.09)
**Anova p value**		< 0.001		< 0.001		< 0.001
**Tumour stage**						
*1*	794	20.1 (22.01)	684	16.1 (19.60)	588	12.6 (16.78)
*2*	484	26.0 (24.31)	427	26.3 (26.38)	365	15.9 (19.77)
*3*	391	27.6 (25.80)	349	29.9 (25.55)	316	17.6 (20.51)
*4*	1176	30.5 (26.29)	999	38.4 (27.15)	920	20.5 (20.65)
**Anova p value**		< 0.001		< 0.001		< 0.001
**Cancer site**						
*Oral cavity*	709	35.0 (25.66)	652	27.2 (25.13)	544	18.6 (20.41)
*Oropharynx*	1120	30.0 (25.82)	959	39.9 (27.01)	906	20.8 (20.91)
*Larynx + Hypopharynx*	710	18.3 (21.31)	589	18.0 (21.17)	516	11.4 (16.45)
*Thyroid*	185	8.2 (11.22)	154	10.4 (17.29)	219	8.7 (14.64)
*Minor and major salivary glands*	131	18.0 (20.91)	111	27.5 (26.37)	101	18.5 (18.66)
**Anova p value**		< 0.001		< 0.001		< 0.001
**HPV16 E6**						
*Positive*	1730	25.7 (25.11)	1497	24.6 (25.28)	1312	16.2 (20.17)
*Negative*	763	27.5 (24.70)	647	38.6 (26.40)	614	19.2 (19.57)
*Unknown*	362	27.5 (25.80)	321	29.6 (27.48)	270	17.9 (18.38)
**Anova p value**		0.623		0.102		0.143

*as measured by EORTC H&N-35

1 “Surgery only” includes 1 patient who had surgery and chemotherapy; “Chemoradiotherapy only” includes 5 patients who only had chemotherapy

By cancer site, at baseline (pre-treatment) the highest prevalence of pain was in patients with oral cavity cancers (48.3%) and the lowest prevalence in thyroid cancer (26.3%). At 4 months, patients with oral cavity cancer experienced slightly less pain (47.3%), whereas all other cancers saw an increase in pain, with over half (53.4%) of oropharyngeal cancer patients experiencing clinically-important pain. At 12 months, every group saw a decline in pain levels with oral, oropharyngeal, and thyroid cancers falling to slightly below baseline levels (38.1%, 34.4% and 24.8% respectively).

Examining pain prevalence by treatment received by 4 months, showed that over half of patients who had multimodal treatment were experiencing clinically-important pain (chemoradiotherapy, 52.6%; surgery with radiotherapy, 57.0%; surgery with chemoradiotherapy, 57.0%) compared to slightly fewer among those who had a single treatment modality. At 12 months, the treatment groups varied relatively little in prevalence. Those diagnosed with stage IV disease had the highest prevalence of pain at all time points. Patterns of prevalence by HPV status varied over time.

The prevalence of clinically-important pain by other socio-demographic, lifestyle and clinical factors at each time point can be seen in Supplementary Table 2. Prevalence was slightly higher in women at each time point, but differences did not reach statistical significance.

### HN-specific pain at each time point

The mean scores followed a similar pattern to general pain, namely an increase from baseline to 4 months (26.39 [standard deviation (SD) 25.10] to 28.89 [SD 26.55]), which was then followed by a decrease to below baseline at 12 months (17.24 [SD 19.83]) (Table 2).

Examining the cohort by HN-specific pain showed similar results and followed largely the same pattern as clinically-important general pain by site, stage, treatment and HPV status. The mean HN-specific pain score was significantly higher in women at 12 months, but not at earlier time points. Mean scores for other clinical and socio-demographic characteristics are summarised in Supplementary Table 3.

### Factors associated with clinically-important pain over time

In the multivariable mixed logistic regression model, the odds of having clinically-important pain were 26% lower at 12 months than at baseline (OR = 0.74, 95%CI 0.64 to 0.87) (Table 3).

**Table 3:** Multivariable logistic mixed regression results: clinically-important pain*. Multivariable odds ratios (OR), 95% confidence intervals (95% CI), p values and Wald test p values**

	OR (95% CI)	P value	Wald test p value
**Time point**			< 0.001
*T1-baseline*	1		
*T2-4 months*	1.67 (1.43 1.94)	< 0.001	
*T3-12 months*	0.74 (0.64 0.87)	< 0.001	
**Age at date of consent (years)**			< 0.001
*< 50*	1		
*50–64*	0.79 (0.60 1.05)	0.100	
$\ge$ *65*	0.54 (0.40 0.73)	< 0.001	
**Depression** ^1^			< 0.001
*No*	1		
*Yes*	8.12 (6.23 10.57)	< 0.001	
**Smoking status**			0.02
*Never*	1		
*Ex*	1.45 (1.15 1.83)	0.002	
*Current*	1.67 (1.22 2.29)	< 0.001	
*Unknown*	1.84 (1.25 2.69)	0.002	
**Deprivation quintile**			< 0.001
*Least deprived (1)*	1		
*2*	1.13 (0.83 1.52)	0.437	
*3*	1.43 (1.08 1.91)	0.014	
*4*	1.47 (1.07 2.01)	0.016	
*Most deprived (5)*	2.17 (1.59 2.96)	< 0.001	
*Unknown*	1.89 (1.30 2.73)	< 0.001	
**Tumour stage**			< 0.001
*1*	1		
*2*	1.44 (1.08 1.92)	0.012	
*3*	1.87 (1.36 2.55)	< 0.001	
*4*	1.92 (1.47 2.50)	< 0.001	
**Comorbidity**			< 0.001
*No co-morbidity*	1		
*Mild decompensation*	1.84 (1.49 2.28)	< 0.001	
*Moderate/severe decompensation*	3.38 (2.59 4.41)	< 0.001	
*Unknown*	3.03 (1.57 5.82)	< 0.001	
**Cancer site**			< 0.001
*Oral cavity*	1		
*Oropharynx*	1.02 (0.75 1.38)	0.901	
*Larynx + Hypopharynx*	0.55 (0.43 0.72)	< 0.001	
*Thyroid*	0.35 (0.23 0.54)	< 0.001	
*Minor and major salivary glands*	0.73 (0.46 1.16)	0.183	
**HPV16 E6**			< 0.001
*Negative*	1		
*Positive*	0.62 (0.46 0.83)	0.002	
*Unknown*	1.27 (0.96 1.70)	0.101	

*as measured by EORTC QLQC-30

**The variables refer to baseline with the exception of time point

1 Score of ≥ 8 on the HADS scale

Over 12-months follow-up, increased age reduced the odds of clinically-important pain, with those over 65 years old having 46% lower odds (OR = 0.54, 95%CI 0.40 to 0.73) compared to those under 50. Those living in more (versus least) deprived communities had 2.17 higher odds (OR = 2.17, 95%CI 1.59 to 2.96).

In terms of lifestyle factors, current smokers at baseline had 67% increased odds (OR = 1.67, 95%CI 1.22 to 2.29) of having clinically-important pain compared to those who never smoked; alcohol intake did not increase the odds of having pain when adjusted for other factors.

In terms of clinical variables, depression at baseline was associated with 8.12-fold increase (OR = 8.12, 95%CI 6.23 to 10.57) of having clinically-important pain. For comorbidity, those with moderate or severe decompensation had an OR of 3.38 (95%CI 2.59 to 4.41) compared to those with no comorbidities.

For cancer-related variables, the odds of clinically-important pain were higher in those with stage 2, 3 or 4 disease compared to stage 1, with stage 4 having 92% increased odds compared to stage 1 disease (OR = 1.92, 95%CI 1.47 to 2.50). Those with oral cavity cancer had the highest odds of clinically-important pain compared with other cancers, and thyroid, and larynx/hypopharynx cancer had the lowest odds (OR = 0.35, 95%CI 0.23 to 0.54 and OR = 0.55, 95%CI 0.43 to 0.72 respectively). Participants with HPV positive cancers had 38% lower odds of experiencing clinically-important pain (OR = 0.62, 95%CI 0.46 to 0.83).

### Factors associated with HN-specific pain over time

In the multivariable model, the HN-specific pain scores increased significantly at 4 months (Coef = 2.57, 95%CI 1.36 to 3.78) and decreased to below baseline levels at 12 months (Coef=-9.32, 95%CI -10.56 to -8.06) (Table 4).

**Table 4:** Multivariable linear mixed regression results – head and neck specific pain*. Coefficient, 95% confidence intervals (95% CI), P values and Wald test P values

	Coefficient (95% CI)	P value	Wald test p value
**Time point**			< 0.001
*T1-baseline*	Reference		
*T2-4 months*	2.57 (1.36 3.78)	< 0.001	
*T3-12 months*	-9.32 (-10.56 -8.06)	< 0.001	
**Age at date of consent (years)**			< 0.001
*< 50*	Reference		
*50–64*	-2.51 (-4.11 -0.90)	< 0.001	
$\ge$ *65*	-6.32 (-8.05 -4.59)	< 0.001	
**Depression**^**1**^			< 0.001
*No*	Reference		
*Yes*	12.28 (10.89 13.66)	< 0.001	
**Smoking status**			< 0.001
*Never*	Reference		
*Ex*	1.99 (0.70 3.28)	< 0.001	
*Current*	7.07 (5.32 8.82)	< 0.001	
*Unknown*	5.06 (2.88 7.24)	< 0.001	
**Treatment received**^**2**^			< 0.001
*Surgery*	Reference		
*Chemoradiotherapy*	8.02 (5.96 10.87)	< 0.001	
*Radiotherapy*	4.87 (2.89 6.85)	< 0.001	
*Surgery and radiotherapy*	10.52 (8.69 12.35)	< 0.001	
*Surgery and chemoradiotherapy*	6.17 (3.82 8.52)	< 0.001	
**Tumour stage**			< 0.001
*1*	Reference		
*2*	2.61 (0.98 4.25)	0.002	
*3*	4.52 (2.64 6.40)	< 0.001	
*4*	5.55 (3.85 7.25)	< 0.001	
**Comorbidity index**			< 0.001
*No co-morbidity*	Reference		
*Mild decompensation*	2.66 (1.45 3.86)	< 0.001	
*Moderate/severe decompensation*	5.56 (4.05 7.06)	< 0.001	
*Unknown*	6.85 (3.01 10.70)	< 0.001	
**Tumour site**			< 0.001
*Oral cavity*	Reference		
*Oropharynx*	-1.87 (-3.83 0.09)	0.061	
*Larynx + Hypopharynx*	-13.37 (-15.20 -11.59)	< 0.001	
*Thyroid*	-15.71 (-18.09 -13.33)	< 0.001	
*Minor and major salivary glands*	-7.48 (-10.16 -4.80)	< 0.001	
**HPV16 E6**			0.006
*Negative*	Reference		
*Positive*	-2.86 (-4.57 -1.15)	0.001	
*Unknown*	0.17 (-1.48 1.81)	0.841	

*The variables refer to baseline with the exception of time point and treatment (which refers to 4 months); pain assessed using EORTC H&N35

1 Score of ≥ 8 on the HADS scale

2 “Surgery only” includes 1 patient who had surgery and chemotherapy; “Chemoradiotherapy only” includes 5 patients who only had chemotherapy

Over the 12 months follow-up, patients under 50 experienced the highest levels of HN-specific pain and scores decreased with increased age. Current smokers scored 7.07 points (95%CI 5.32 to 8.82) higher on the HN-specific pain scale than non-smokers.

Those with depression scored 12.28 (95%CI 10.89 to 13.66) points higher for HN-specific pain than those without depression. Individuals with moderate or severe decompensation scored 5.56 (95%CI 3.85 to 7.25) higher than those with no comorbidities.

Several cancer-related variables also impacted the HN-specific pain scores. Scores were highest in individuals with stage 4 disease when compared with stage 1 tumours (Coef = 5.55, 95%CI 3.85 to 7.25). Those with oral cavity cancer scored highest compared to other types of HNC; thyroid, laryngeal and hypopharynx cancer patients had the lowest scores (Coef=-15.71, 95%CI -18.09 to -13.33 and − 13.39, 95%CI -15.20 to -11.59 respectively). In terms of treatment, participants who only had surgery had the lowest pain scores, while those who underwent multimodal treatment scored higher. Compared with surgery alone, the surgery combined with radiotherapy group scored 10.91 (95%CI 8.33 to 13.49) higher and the chemoradiotherapy group scored 8.40 points (95% CI 5.53 to 11.26) higher. Participants with HPV positive tumours scored 2.60 points lower (95%CI -4.57 to -1.15) than those with HPV negative tumours.

## Discussion

This study is the first large-scale longitudinal investigation of both clinically-important general pain and HN-specific pain in HNC patients and predictors of this pain over time. For both pain outcomes, we found that cancer-related factors such as tumour stage, tumour site, HPV status and treatment were associated with pain over 12 months. Younger age, depression, deprivation, smoking, and comorbidities also had an association with higher levels of pain.

### Temporal trends in pain

One of the key findings was the high prevalence of general pain in HNC; the percentage reporting clinically-important pain was high at baseline (41%), increased to almost half (48%) at 4 months, then declined, but only to 36%, at 12 months. HN-specific pain scores also followed a similar pattern. For both outcomes, the 12-month levels were lower than at baseline, but they were still high. The baseline prevalence could, perhaps, be explained by the aetiology of the pain in HNC and the location of the tumour. Baseline pain in HNC may be related to tumour invasion of the bones and soft tissues, benign inflammation, superimposed infections, or due to direct nerve involvement [8]. In many cancers, systemic inflammatory response has been demonstrated to upregulate the innate immune response, which in turn causes a systemic inflammatory response thus resulting in more significant levels of pain [36]. Head and neck squamous cell carcinomas are the most common type of HNC, and they are highly inflammatory and aggressive tumours, which could be contributing to the high levels of pain these patients experience [37, 38].

Our findings of high pain levels at 4 months are consistent with other studies [7, 39] and are likely explained by proximity to treatment, especially in those who received multi-modal treatments. Notably chemo- and radiotherapy are associated with pro-inflammatory cytokines and a systemic inflammatory reaction, thus increasing a pain response [40].

The relative reduction in pain at 12 months, is, perhaps, explained by the reduction of tumour-related pain factors such as inflammation or direct nerve involvement [8]. Our findings appear to contradict a 2010 systematic review which suggested pain does not return to baseline levels and may be higher post- than pre-treatment [39]. However, the studies included in that review had comparatively small sample sizes (*N* = 20 to 357), and varying times from diagnosis (and baseline) to treatment.

### Clinical predictors of pain

Patients having multimodal treatment had significantly higher HN-specific pain scores compared to those who had surgery alone. This is consistent with previous reports [7, 39] and could be explained by the combined side-effects of multiple treatments (e.g., the combination of radiotherapy-induced oral mucositis, damage to sensory nerves in the region during surgery, or increased rates of infections or peripheral neuropathy following chemotherapy). Interestingly, this pattern was not seen on the general pain scale, although it is possible that patients may have experience general pain, but not at a level meeting the threshold for clinical importance.

There are mixed reports of the impact of cancer stage on pain [7, 15]. Our study found that patients with advanced disease at diagnosis had increased odds of both clinically-important general pain and higher HN-specific pain scores. Advanced disease stage indicates either a larger tumour and/or local metastatic disease. Lymph node metastases and large head and neck tumours can impinge on adjacent tissues, obstruct blood vessels, and increase tumour-induced cytokines, activating visceral or somatic nociceptors, thus leading to increased levels of pain [1, 10].

Participants with oral cavity cancer had the highest odds of clinically-important general pain and HN-specific pain scores, and patients with thyroid and larynx/hypopharynx cancer had the lowest odds and scores. This is consistent with smaller, cross-sectional studies [1, 7]. Higher levels of oral cavity cancer pain could partially be explained by the greater prevalence of oral mucosal damage, due to both the disease and its treatment [41, 42].

We also found that patients with HPV positive tumours reported lower pain scores compared to patients with HPV negative tumours, after adjusting for other cancer-related variables. This appears to be a novel finding and explanations are not obvious. Previous studies found that HPV positive tumours more commonly presented with neck mass rather than pain [43]. However, HPV can infect the stratified squamous epithelia in the mouth, throat, and respiratory tract [44]; this, in turn, could affect the nociceptors that have been found to play a role in HNC pain perception [1]. Another possibility is that the finding is driven by inflammation. Xiao et al. reported higher levels of systemic inflammation (measured by C-reactive protein, soluble tumour necrosis factor receptor-2, and interleukin-6 protein) in patients with tumours not related to HPV [45].

### Other predictors of pain

We identified several other pain predictors in this study. The strongest relationship was identified in patients with depression at baseline, who had an 8-fold increase in their odds of having clinically-important pain. This supports findings from some other small studies [46, 47] but contradicts others [48]. Uniquely, our study demonstrates this relationship persists over 12 months after adjusting for other variables. Depression has previously been reported as an independent risk factor for the onset of chronic pain in non-cancer patients, and in a separate study, the prevalence of pain in patients with depression was 59% [49, 50]. Additionally, in oncological patients, Derogatis et al. showed that those with diagnosed psychiatric conditions (including depression) were more likely to report significant pain compared to those with no psychiatric diagnosis [51]. Pain and depression are frequently encountered in clinical practice and their coexistence tends to further aggravate the severity of both disorders [52], which could provide a potential explanation for our findings.

Other non-cancer characteristics that were found to be associated with pain were age, comorbidities, and smoking. While all three variables were fitted simultaneously in the models, they are inter-related in the study population making it hard to be confident that these are truly independent effects. Similar to previous research, younger people (< 50) reported more pain than those aged $\ge$65 [7]. Gagliese et al. suggest that younger people with cancer pain are less likely to employ strategies to manage the pain [53]. Others report more frequent cancer pain flares in younger people [54]. The EORTC questionnaires used here focus on pain in the last week and perhaps preferentially capture these flares better than chronic, but more stable, pain. Contrary to previous reports [55–57], we identified a relationship between comorbidity and increased pain, assessed using the ACE-27 scoring system. The ACE-27 covers a broad range of conditions, and it is unclear whether specific co-morbidities might contribute to the pain reported. Others have suggested a relationship between smoking and pain [14, 58] and our results demonstrate that this persists over 12 months. In terms of other lifestyle factors, in univariable analyses, more frequent alcohol consumption was associated with lower prevalence of clinically-important general pain; while this association no long reached statistical significance in the multivariable model, it is intriguing. Others have reported insufficient pain control in people with cancer [59] and it is possible that our univariable findings reflect use of alcohol to self-medicate for uncontrolled cancer pain.

### Implications

These findings show that a significant proportion of HNC patients suffer from pain at diagnosis and after treatment; it seems likely that this, in turn, has a substantial impact on QoL.

The high burden of pain in HNC patients with depression is particularly concerning, and clinical teams should put a greater emphasis on both diagnosis and treatment of depression in this population. Screening questionnaires for depression, such as the HADS, could be used more frequently and HNC multi-disciplinary teams could be expended to include clinical psychologists more routinely.

Systematic pain screening could also help to identify who could benefit from a pain management plan at an earlier stage. Appropriate analgesia should be a priority, and Ren et al. [47] have previously shown that while 11.6% of patients with HNC reported neuropathic pain, only 3.8% were taking psychotropic medications targeting this kind of pain. It is therefore important for clinicians to identify the type of pain these patients experience and prescribe their analgesia accordingly.

Other, non-medical strategies for pain control should also be utilised, such as complementary therapies [60]. Several studies have shown that physical activity significantly improves specific HNC-related pain [61–63]. Larger scale randomised studies of interventions to encourage and support physical activity are warranted to explore the potential improvements in QoL and pain in this patient group.

### Strengths and limitations

There was wide variability in the reporting of prevalence of cancer pain in HNC patients in previous studies; a 2012 systematic review reported prevalence ranged from 9 to 98% [7]. This variability is caused by a number of issues, including variations in: study design; distributions of age, primary site, and stages of included patients; treatment modalities received; definition of pain used; and method(s) of pain evaluation. The review highlighted the need for high quality epidemiological studies. In response, we conducted the largest cohort study to date and reported on pre- and post- treatment HNC pain. We used two EORTC instruments to capture both general and HN-specific pain, reported descriptive data on pain levels in different patient subgroups, and applied robust statistical methods to investigate trends and associations.

There are some limitations to our study. Although widely used, the pain subscales contain only two general and four HN-specific questions, and it is possible these do not capture all manifestations of pain in HNC patients. It is also worth noting that the instruments only capture pain experienced in the last week. Moreover, it is difficult to determine whether the patients’ pain is fully due to the cancer or other non-cancer causes (e.g., arthritis or other comorbid conditions).

The overall prevalence of general pain and HN-specific pain scores are also likely underestimated in our analysis. Patients with more advanced cancer – which was associated here with more pain - were underrepresented in the HN5000 cohort, and our study population.

## Conclusions

Over a third of HNC patients experience clinically-important pain at 12 months post-diagnosis. Socio-demographic, cancer-specific, and other clinical variables predicted likelihood of clinically-important general pain and HN-specific pain scores over 12 months. These findings show that there is a need for additional interventions focusing on addressing this problem before, during and after cancer treatment.

## Electronic supplementary material

Below is the link to the electronic supplementary material.

Supplementary Material 1

## Data Availability

The data that support the findings of this study are available from Head and Neck 5000. Further information may be found on the Head and Neck 5000 website: https://www.headandneck5000.org.uk/ information-for-researchers.
