# Supplementary material for: Cancer-related pain in head and neck cancer survivors: longitudinal findings from the Head and Neck 5000 clinical cohort
Source: J Cancer Surviv. 2024 Feb 29;19(4):1313–24. doi: 10.1007/s11764-024-01554-x (PMC12283790; doi:10.1007/s11764-024-01554-x)
Supplement: Supplementary file 1 — Supplementary Material 1 [file 11764_2024_1554_MOESM1_ESM.docx]

***Supplementary Table 1. Baseline characteristics of the study population. Includes individuals with non-recurrent HNC who had received treatment (N =*2,870 and 2,855 for the QLQ-C30 and H&N35 respectively*).***

|  | Participants in analyses of general pain (QLQ-C30)  N (%) | Participants in analyses of HN-specific pain (H&N35)  N (%) |
| --- | --- | --- |
| **Age at date of consent (years)** |  |  |
| *<50* | 446 (15.5) | 443 (15.6) |
| *50-64* | 1355 (47.2) | 1349 (74.3) |
| $\geq$*65* | 1069 (37.3) | 1063 (37.2) |
|  |  |  |
| **Sex** |  |  |
| *Male* | 2042 (71.2) | 2030 (71.1) |
| *Female* | 828 (28.9) | 825 (28.9) |
|  |  |  |
| **Income (£)** |  |  |
| *<3999-7999* | 372 (15.4) | 374 (15.5) |
| *8000 - 17999* | 709 (29.4) | 707 (29.3) |
| *18000 – 28999* | 527 (21.8) | 522 (12.7) |
| *29000 – 35000+* | 808 (33.4) | 807 (33.5) |
| *Unknown* | 454 (15.8) | 445 (15.6) |
|  |  |  |
| **Education** |  |  |
| *Primary/Secondary* | 1192 (41.5) | 1187 (41.6) |
| *Tertiary* | 1439 (50.1) | 4133 (50.2) |
| *Unknown* | 239 (8.3) | 235 (8.2) |
|  |  |  |
| **Depression^1^** |  |  |
| *No* | 2340 (82.0) | 2329 (82.0) |
| *Yes* | 513 (18.0) | 510 (18.0) |
|  |  |  |
| **Alcohol consumption (days per week)** |  |  |
| *0* | 750 (26.1) | 748 (26.2) |
| *1-3* | 655 (22.8) | 650 (22.8) |
| *3-7* | 1253 (43.7) | 1245 (43.6) |
| *Unknown* | 212 (7.4) | 212 (7.4) |
|  |  |  |
| **Smoking status** |  |  |
| *Never* | 675 (23.5) | 674 (23.6) |
| *Ex* | 1493 (52.0) | 1484 (52.0) |
| *Current* | 484 (16.9) | 480 (16.8) |
| *Unknown* | 218 (7.6) | 217 (7.6) |
|  |  |  |
| **Marital status** |  |  |
| *Married* | 1919 (66.9) | 1913 (67.0) |
| *Other* | 850 (29.6) | 843 (30.0) |
| *Unknown* | 101 (3.5) | 99 (3.5) |
|  |  |  |
| **Deprivation quintile** |  |  |
| *Least Deprived (1)* | 525 (18.3) | 524 (18.4) |
| *2* | 511 (17.8) | 512 (17.9) |
| *3* | 614 (21.4) | 609 (21.3) |
| *4* | 460 (16.0) | 455 (15.9) |
| *Most deprived (5)* | 507 (17.7) | 503 (17.6) |
| *Unknown* | 253 (8.8) | 252 (8.8) |
|  |  |  |
| **Treatment received^2^** |  |  |
| *Surgery* | 845 (29.4) | 842 (29.5) |
| *Chemoradiotherapy* | 787 (27.4) | 783 (27.4) |
| *Radiotherapy* | 540 (18.8) | 538 (18.8) |
| *Surgery and radiotherapy* | 421 (14.7) | 416 (14.6) |
| *Surgery and chemoradiotherapy* | 277 (9.7) | 276 (9.7) |
|  |  |  |
| **Comorbidity index** |  |  |
| *No co-morbidity* | 1311 (45.7) | 1306 (45.7) |
| *Mild decompensation* | 952 (33.2) | 946 (33.1) |
| *Moderate/severe decompensation* | 511 (19.2) | 547 (19.2) |
|  |  |  |
| **Cancer site** |  |  |
| *Oral cavity* | 716 (25.0) | 709 (24.8) |
| *Oropharynx* | 1123 (39.1) | 1120 (39.2) |
| *Larynx + Hypopharynx* | 713 (24.8) | 710 (24.9) |
| *Thyroid* | 186 (6.5) | 185 (6.5) |
| *Minor and major salivary glands* | 132 (4.6) | 131 (4.6) |
|  |  |  |
| **Tumour stage** |  |  |
| *1* | 797 (27.9) | 794 (27.9) |
| *2* | 485 (17.0) | 484 (17.0) |
| *3* | 393 (14.0) | 391 (13.7) |
| *4* | 1814 (41.4) | 1176 (41.3) |
|  |  |  |
| **HPV16 E6** |  |  |
| *Positive* | 1741 (60.7) | 1730 (60.6) |
| *Negative* | 765 (26.7) | 763 (26.7) |
| *Unknown* | 364 (12.7) | 362 (12.7) |
|  |  |  |
| **Primary tumour side^3^** |  |  |
| *Unilateral* | 2583 (90.0) | 2569 (90.0) |
| *Bilateral* | 278 (9.7) | 277 (9.7) |

***1 – Scores of ≥8 on the HADS scale***

***2 – “Surgery only” includes 1 patient who had surgery and chemotherapy; “Chemoradiotherapy only” includes 5 patients who only had chemotherapy***

***3 – Bilateral group includes midline, bilateral, left/midline and right/midline groups***

***Supplementary table 2. Prevalence of clinically-important general pain* at baseline, 4 months, and 12 months, by sociodemographic, lifestyle, and other clinical variables. Number who completed subscale (N), number who scored in range for clinically-important pain (n) and percentages (%)***

|  | Baseline | | 4 Months | | 12 Months | |
| --- | --- | --- | --- | --- | --- | --- |
|  | N (n) | % clinically-important | N(n) | % clinically-important | N(n) | % clinically-important |
| **Overall** | 2870 (1175) | 40.9 | 2469 (1174) | 47.6 | 2205 (782) | 35.5 |
| **Age at date of consent (years)** | | | | | | |
| *<50* | 446 (192) | 43.1 | 345 (163) | 47.3 | 284 (100) | 35.2 |
| *50-64* | 1355 (569) | 42.0 | 1159 (601) | 51.9 | 1059 (410) | 38.7 |
| $\geq$*65* | 1069 (414) | 38.7 | 965 (410) | 42.5 | 862 (272) | 31.6 |
| **Chi2 p value** |  | 0.165 |  | <0.001 |  | 0.005 |
|  |  |  |  |  |  |  |
| **Sex** | | | | | | |
| *Male* | 2042 (820) | 40.2 | 1765 (832) | 47.1 | 1574 (543) | 34.5 |
| *Female* | 828 (355) | 42.9 | 704 (342) | 48.6 | 631 (239) | 37.9 |
| **Chi2 p value** |  | 0.180 |  | 0.518 |  | 0.134 |
|  |  |  |  |  |  |  |
| **Income** | | | | | | |
| *<3999-7999* | 372 (189) | 50.8 | 266 (166) | 62.4 | 243 (130) | 53.5 |
| *8000 - 17999* | 709 (329) | 46.4 | 540 (258) | 47.8 | 503 (190) | 37.8 |
| *18000 – 28999* | 527 (188) | 35.7 | 436 (186) | 42.7 | 381 (114) | 29.9 |
| *29000 – 35000+* | 808 (250) | 30.9 | 674 (266) | 39.5 | 626 (147) | 23.5 |
| **Chi2 p value** |  | <0.001 |  | <0.001 |  | <0.001 |
|  |  |  |  |  |  |  |
| **Education** |  |  |  |  |  |  |
| *Primary/Secondary* | 1192 (521) | 43.7 | 904 (437) | 48.3 | 801 (312) | 39.0 |
| *Tertiary* | 1439 (534) | 37.1 | 1156 (514) | 44.5 | 1063 (325) | 30.6 |
| *Unknown* | 239 (120) | 50.2 | 409 (223) | 54.5 | 341 (145) | 42.5 |
| **Chi2 p value** |  | <0.001 |  | 0.002 |  | <0.001 |
|  |  |  |  |  |  |  |
| **Depression^1^** | | | | | | |
| *No* | 2340 (787) | 33.6 | 1065 (773) | 57.9 | 1658 (481) | 29.1 |
| *Yes* | 513 (380) | 74.1 | 339 (244) | 42.1 | 313 (200) | 36.9 |
| **Chi2 p value** |  | <0.001 |  | <0.001 |  | <0.001 |
|  |  |  |  |  |  |  |
| **Alcohol consumption (days per week)** | | | | | | |
| *0* | 750 (331) | 44.1 | 562 (266) | 47.3 | 534 (214) | 40.1 |
| *1-3* | 655 (230) | 35.1 | 532 (225) | 42.3 | 464 (134) | 28.9 |
| *3-7* | 1253 (508) | 40.5 | 986 (467) | 47.4 | 883 (292) | 33.1 |
| *Unknown* | 212 (106) | 50.0 | 389 (216) | 55.5 | 324 (142) | 43.8 |
| **Chi2p value** |  | <0.001 |  | 0.001 |  | <0.001 |
|  |  |  |  |  |  |  |
| **Smoking status** | | | | | | |
| *Never* | 675 (227) | 33.6 | 547 (208) | 38.0 | 509 (135) | 62.5 |
| *Ex* | 1493 (616) | 41.3 | 1182 (555) | 47.0 | 1079 (370) | 34.3 |
| *Current* | 484 (235) | 48.6 | 335 (191) | 57.0 | 275 (136) | 49.5 |
| *Unknown* | 218 (97) | 44.5 | 405 (220) | 54.3 | 342 (141) | 41.2 |
| **Chi2 P value** |  | <0.001 |  | <0.001 |  | <0.001 |
|  |  |  |  |  |  |  |
| **Marital status** | | | | | | |
| *Married* | 1919 (750) | 39.1 | 1533 (693) | 45.2 | 1395 (451) | 32.3 |
| *Other* | 850 (381) | 44.8 | 625 (312) | 49.9 | 557 (220) | 39.5 |
| *Unknown* | 101 (44) | 43.6 | 311 (169) | 54.3 | 253 (111) | 43.9 |
| **Chi2 p value** |  | 0.016 |  | 0.005 |  | <0.001 |
|  |  |  |  |  |  |  |
| **Deprivation quintile** | | | | | | |
| *Least Deprived (1)* | 525 (178) | 33.9 | 483 (184) | 38.1 | 444 (108) | 24.3 |
| *2* | 511 (176) | 34.4 | 453 (196) | 43.3 | 426 (122) | 28.6 |
| *3* | 614 (246) | 40.1 | 517 (242) | 46.8 | 468 (171) | 36.5 |
| *4* | 460 (205) | 44.6 | 378 (188) | 49.7 | 330 (135) | 40.9 |
| *Most deprived (5)* | 507 (254) | 50.1 | 419 (246) | 58.7 | 345 (168) | 48.7 |
| *Unknown* | 253 (116) | 45.9 | 219 (118) | 53.9 | 192 (78) | 40.6 |
| **Chi2 p value** |  | <0.001 |  | <0.001 |  | <0.001 |
|  |  |  |  |  |  |  |
| **Treatment received^2^** | | | | | | |
| *Surgery* | 845 (325) | 38.5 | 752 (293) | 39.0 | 644 (229) | 35.6 |
| *Chemoradiotherapy* | 787 (332) | 42.2 | 666 (350) | 52.6 | 617 (213) | 34.5 |
| *Radiotherapy* | 540 (205) | 38.0 | 435 (180) | 41.4 | 395 (139) | 35.2 |
| *Surgery and radiotherapy* | 421 (191) | 45.4 | 379 (216) | 57.0 | 345 (125) | 36.2 |
| *Surgery and chemoradiotherapy* | 277 (122) | 44.0 | 237 (135) | 57.0 | 204 (76) | 37.3 |
| **Chi2 p value** |  | 0.057 |  | <0.001 |  | 0.960 |
|  |  |  |  |  |  |  |
| **Comorbidity index** | | | | | | |
| *No co-morbidity* | 1311 (437) | 33.3 | 1140 (464) | 40.7 | 1014 (274) | 27.0 |
| *Mild decompensation* | 952 (399) | 41.9 | 825 (413) | 50.1 | 760 (299) | 39.3 |
| *Moderate/severe decompensation* | 551 (314) | 57.0 | 458 (273) | 59.7 | 390 (189) | 48.5 |
| *Unknown* | 56 (25) | 44.6 | 46 (24) | 52.2 | 41(20) | 48.8 |
| **Chi2 p value** |  | <0.001 |  | <0.001 |  | <0.001 |
|  |  |  |  |  |  |  |
| **Cancer site** | | | | | | |
| *Oral cavity* | 716 (346) | 48.3 | 655 (310) | 47.3 | 548 (209) | 38.1 |
| *Oropharynx* | 1123 (484) | 43.1 | 958 (512) | 53.4 | 911 (313) | 34.4 |
| *Larynx + Hypopharynx* | 713 (254) | 35.6 | 590 (257) | 43.6 | 518 (192) | 37.1 |
| *Thyroid* | 186 (49) | 26.3 | 155 (46) | 29.7 | 129 (32) | 24.8 |
| *Minor and major salivary glands* | 132 (42) | 31.8 | 111 (49) | 44.1 | 99 (36) | 36.4 |
| **Chi2 p value** |  | <0.001 |  | <0.001 |  | 0.056 |
|  |  |  |  |  |  |  |
| **Tumour stage** | | | | | | |
| *1* | 797 (275) | 34.5 | 685 (245) | 35.8 | 590 (189) | 32.0 |
| *2* | 485 (205) | 42.3 | 426 (197) | 46.2 | 367 (133) | 36.2 |
| *3* | 393 (159) | 40.5 | 349 (177) | 50.7 | 318 (127) | 39.9 |
| *4* | 1184 (532) | 44.9 | 1003 (552) | 55.0 | 923 (330) | 35.8 |
| **Chi2 p value** |  | <0.001 |  | <0.001 |  | 0.114 |
|  |  |  |  |  |  |  |
| **HPV16 E6** | | | | | | |
| *Positive* | 1741 (713) | 41.0 | 1500 (674) | 44.9 | 1318 (479) | 36.3 |
| *Negative* | 765 (288) | 37.7 | 647 (324) | 50.1 | 617 (194) | 31.4 |
| *Unknown* | 364 (174) | 47.8 | 322 (176) | 54.7 | 270 (109) | 40.4 |
| **Chi2 p value** |  | 0.005 |  | 0.002 |  | 0.022 |
|  |  |  |  |  |  |  |
| **Primary tumour side^3^** | | | | | | |
| *Unilateral* | 2583 (1058) | 41.0 | 2228 (1052) | 47.2 | 1994 (698) | 35.0 |
| *Bilateral* | 278 (114) | 41.0 | 235 (118) | 50.2 | 206 (80) | 38.8 |
| **T-test p value** |  | 0.897 |  | 0.439 |  | 0.063 |

***1 – Scores of ≥8 on the HADS scale***

***2 – “Surgery only” includes 1 patient who had surgery and chemotherapy; “Chemoradiotherapy only” includes 5 patients who only had chemotherapy***

***3 – Bilateral group includes midline, bilateral, left/midline and right/midline groups***

****as measured by EORTC QLQC-30***

***Supplementary Table 3 Head and neck specific pain*: mean scores (standard deviation) at baseline, 4 months, and 12 months, by sociodemographic, lifestyle, and other clinical variables. Number of participants who completed subscale (N).***

| **Explanatory variable** | Baseline | | 4 Months | | 12 Months | |
| --- | --- | --- | --- | --- | --- | --- |
|  | N | Mean score (sd) | N | Mean score (sd) | N | Mean score (sd) |
| **Overall** | 2855 | 26.39 (25.10) | 2465 | 28.89 (26.55) | 2196 | 17.24 (19.83) |
| **Age at date of consent (years)** | | | | | | |
| *<50* | 443 | 28.13 (27.45) | 346 | 29.45 (27.24) | 284 | 18.56 (20.07) |
| *50-64* | 1349 | 28.33 (25.50) | 1160 | 33.54 (26.95) | 1056 | 19.47 (20.81) |
| $\geq$*65* | 1063 | 23.19 (23.18) | 959 | 23.45 (24.66) | 856 | 14.05 (18.02) |
| **Anova p value** |  | <0.001 |  | 0.01 |  | <0.001 |
|  |  |  |  |  |  |  |
| **Sex** | | | | | | |
| *Male* | 2030 | 26.53 (25.19) | 1762 | 29.11 (26.40) | 1568 | 16.15 (18.83) |
| *Female* | 825 | 26.04 (24.88) | 703 | 28.36 (26.95) | 628 | 19.97 (21.91) |
| **T-test value** |  | 0.68 |  | 0.51 |  | <0.001 |
|  |  |  |  |  |  |  |
| **Income** | | | | | | |
| *<3999-7999* | 374 | 31.42 (27.21) | 266 | 33.36 (29.31) | 241 | 22.30 (22.13) |
| *8000 - 17999* | 707 | 27.59 (26.68) | 541 | 27.69 (26.70) | 502 | 18.25 (21.60) |
| *18000 – 28999* | 522 | 25.21 (23.35) | 434 | 27.18 (25.84) | 379 | 14.95 (17.50) |
| *29000 – 35000+* | 807 | 22.62 (22.85) | 671 | 28.78 (25.22) | 626 | 14.41 (16.28) |
| **Anova p value** |  | <0.001 |  | 0.024 |  | <0.001 |
|  |  |  |  |  |  |  |
| **Education** | | | | | | |
| *Primary/Secondary* | 1187 | 28.17 (25.91) | 902 | 28.64 (27.29) | 798 | 17.51 (21.10) |
| *Tertiary* | 1433 | 24.70 (24.13) | 1154 | 27.97 (25.59) | 1063 | 15.93 (18.05) |
| *Unknown* | 235 | 27.67 (26.15) | 409 | 32.05 (27.41) | 335 | 20.74 (21.60) |
| **Anova p value** |  | 0.023 |  | 0.071 |  | <0.001 |
|  |  |  |  |  |  |  |
| **Depression^1^** | | | | | | |
| *No* | 2329 | 23.27 (23.13) | 1833 | 26.76 (25.71) | 1652 | 14.80 (17.81) |
| *Yes* | 510 | 40.85 (28.57) | 340 | 38.12 (28.69) | 313 | 26.54 (24.41) |
| **T-test p value** |  | <0.001 |  | <0.001 |  | <0.001 |
|  |  |  |  |  |  |  |
| **Alcohol consumption (days per week)** | | | | | | |
| *0* | 748 | 26.05 (25.77) | 562 | 28.13 (26.73) | 535 | 17.71 (21.27) |
| *1-3* | 650 | 23.35 (23.44) | 531 | 26.20 (25.54) | 461 | 14.12 (16.32) |
| *3-7* | 1245 | 27.30 (24.84) | 984 | 28.96 (26.16) | 881 | 16.58 (19.02) |
| **Anova p value** |  | 0.045 |  | 0.567 |  | <0.001 |
|  |  |  |  |  |  |  |
| **Smoking status** | | | | | | |
| *Never* | 674 | 22.81 (23.59) | 549 | 26.86 (25.78) | 508 | 14.74 (17.12) |
| *Ex* | 1484 | 25.46 (24.29) | 1177 | 27.10 (26.06) | 1077 | 15.46 (18.74) |
| *Current* | 480 | 33.95 (27.67) | 336 | 34.57 (27.75) | 274 | 23.74 (23.72) |
| *Unknown* | 217 | 27.10 (25.80) | 403 | 32.18 (27.10) | 337 | 21.40 (21.73) |
| **Anova p value** |  | <0.001 |  | 0.35 |  | <0.001 |
|  |  |  |  |  |  |  |
| **Marital status** | | | | | | |
| *Married/living together* | 1913 | 25.78 (24.67) | 1527 | 28.29 (26.20) | 1392 | 15.83 (18.57) |
| *Other* | 843 | 27.69 (26.03) | 627 | 29.03 (27.16) | 555 | 18.81 (20.93) |
| *Unknown* | 99 | 26.91 (25.02) | 311 | 31.57 (26.96) | 249 | 21.64 (23.02) |
| **Anova p value** |  | 0.185 |  | 0.138 |  | <0.001 |
|  |  |  |  |  |  |  |
| **Deprivation quintile** | | | | | | |
| *Least Deprived (1)* | 524 | 23.22 (23.14) | 480 | 25.63 (24.12) | 441 | 13.90 (16.54) |
| *2* | 512 | 24.52 (24.31) | 451 | 28.23 (27.51) | 424 | 15.40 (18.78) |
| *3* | 609 | 25.94 (23.90) | 516 | 28.25 (26.01) | 468 | 16.95 (19.55) |
| *4* | 455 | 27.65 (26.71) | 380 | 29.97 (27.06) | 331 | 20.49 (21.78) |
| *Most deprived (5)* | 503 | 30.03 (26.36) | 418 | 32.42 (27.66) | 341 | 20.94 (22.08) |
| *Unknown* | 252 | 29.30 (26.80) | 220 | 30.33 (27.17) | 191 | 17.51 (20.28) |
| **Anova p value** |  | <0.001 |  | 0.04 |  | <0.001 |
|  |  |  |  |  |  |  |
| **Treatment received^2^** | | | | | | |
| *Surgery* | 842 | 23.41 (23.31) | 752 | 15.52 (18.97) | 642 | 13.44 (17.74) |
| *Chemoradiotherapy* | 783 | 29.50 (25.67) | 666 | 39.34 (26.42) | 614 | 21.18 (21.18) |
| *Radiotherapy* | 538 | 19.42 (21.62) | 434 | 21.25 (23.73) | 393 | 13.89 (18.59) |
| *Surgery and radiotherapy* | 416 | 32.53 (28.48) | 378 | 39.24 (28.04) | 343 | 19.66 (19.62) |
| *Surgery and chemoradiotherapy* | 276 | 30.93 (26.38) | 235 | 29.56 (26.40) | 204 | 19.74 (21.09) |
| **Anova p value** |  | <0.001 |  | <0.001 |  | <0.001 |
|  |  |  |  |  |  |  |
| **Comorbidity index** | | | | | | |
| *No co-morbidity* | 1306 | 24.17 (23.94) | 1139 | 27.69 (25.54) | 1009 | 15.29 (17.80) |
| *Mild decompensation* | 946 | 26.43 (24.63) | 824 | 28.62 (26.22) | 757 | 18.31 (20.78) |
| *Moderate/severe decompensation* | 547 | 31.59 (27.61) | 465 | 32.01 (28.99) | 389 | 20.04 (22.67) |
| *Unknown* | 56 | 26.44 (26.77) | 46 | 32.91 (29.76) | 41 | 18.97 (20.64) |
| **Anova p value** |  | 0.001 |  | 0.07 |  | <0.001 |
|  |  |  |  |  |  |  |
| **Cancer site** | | | | | | |
| *Oral cavity* | 709 | 34.98 (25.66) | 652 | 27.19 (25.13) | 544 | 18.61 (20.41) |
| *Oropharynx* | 1120 | 30.04 (25.82) | 959 | 39.90 (27.01) | 906 | 20.80 (20.91) |
| *Larynx + Hypopharynx* | 710 | 18.34 (21.31) | 589 | 17.96 (21.17) | 516 | 11.42 (16.45) |
| *Thyroid* | 185 | 8.18 (11.22) | 154 | 10.39 (17.29) | 219 | 8.74 (14.64) |
| *Minor and major salivary glands* | 131 | 17.96 (20.91) | 111 | 27.45 (26.37) | 101 | 18.45 (18.66) |
| **Anova p value** |  | <0.001 |  | <0.001 |  | <0.001 |
|  |  |  |  |  |  |  |
| **Tumour stage** | | | | | | |
| *1* | 794 | 20.12 (22.01) | 684 | 16.11 (19.60) | 588 | 12.62 (16.78) |
| *2* | 484 | 25.96 (24.31) | 427 | 26.28 (26.38) | 365 | 15.87 (19.77) |
| *3* | 391 | 27.61 (25.80) | 349 | 29.89 (25.55) | 316 | 17.61 (20.51) |
| *4* | 1176 | 30.47 (26.29) | 999 | 38.38 (27.15) | 920 | 20.49 (20.65) |
| **Anova p value** |  | <0.001 |  | <0.001 |  | <0.001 |
|  |  |  |  |  |  |  |
| **HPV16 E6** | | | | | | |
| *Positive* | 1730 | 25.68 (25.11) | 1497 | 24.55 (25.28) | 1312 | 16.19 (20.17) |
| *Negative* | 763 | 27.48 (24.70) | 647 | 38.61 (26.40) | 614 | 19.20 (19.57) |
| *Unknown* | 362 | 27.48 (25.80) | 321 | 29.57 (27.48) | 270 | 17.86 (18.38) |
| **Anova p value** |  | 0.623 |  | 0.102 |  | 0.143 |
|  |  |  |  |  |  |  |
| **Primary tumour side^3^** | | | | | | |
| *Unilateral* | 2569 | 26.16 (24.99) | 2222 | 28.84 (26.45) | 1986 | 17.17 (19.76) |
| *Bilateral* | 277 | 28.47 (26.07) | 237 | 29.29 (27.38) | 205 | 17.33 (19.60) |
| **T-Test P value** |  | 0.616 |  | 0.439 |  | 0.022 |

***1 – Scores of ≥8 on the HADS scale***

***2 – “Surgery only” includes 1 patient who had surgery and chemotherapy; “Chemoradiotherapy only” includes 5 patients who only had chemotherapy***

***3 – Bilateral group includes midline, bilateral, left/midline and right/midline groups***

****as measured by the EORTC H&N35***

***Supplementary Figure 1 –Flow diagram showing the study population***

5404 participants included in the HN5000 dataset

451 - tumours other than oral cavity, oropharynx, larynx, hypopharynx, thyroid, and salivary glands

909 did not complete baseline QLQ-C30 questionnaire

924 did not complete baseline QLQ-H&N35 questionnaire

2870 Participants answered the QLQ-C30 baseline questionnaire and completed pain subscale

2855 Participants answered the QLQ-H&N35 baseline questionnaire and completed pain subscale

521 had tumour recurrence at month 4; 601 had tumour recurrence at month 12

52 – did not receive treatment

3779 included in study: non-recurrent oral cavity, oropharynx, larynx, hypopharynx, thyroid, and salivary glands tumours

4901 received initial treatment (curative, palliative or best supportive care)

4953 had cancers of the oral cavity, oropharynx, larynx, hypopharynx, thyroid, and salivary glands tumours
